# The case for eliminating excessive worry as a requirement for generalized anxiety disorder: a cross-national investigation

**DOI:** 10.1017/S003329172400182X

**Published:** 2024-09

**Authors:** Ayelet Meron Ruscio, Madeleine Rassaby, Murray B. Stein, Dan J. Stein, Sergio Aguilar-Gaxiola, Ali Al-Hamzawi, Jordi Alonso, Lukoye Atwoli, Guilherme Borges, Evelyn J. Bromet, Ronny Bruffaerts, Brendan Bunting, Graça Cardoso, Stephanie Chardoul, Giovanni de Girolamo, Peter de Jonge, Oye Gureje, Josep Maria Haro, Elie G. Karam, Aimee Karam, Andrzej Kiejna, Viviane Kovess-Masfety, Sue Lee, Fernando Navarro-Mateu, Daisuke Nishi, Marina Piazza, José Posada-Villa, Nancy A. Sampson, Kate M. Scott, Tim Slade, Juan Carlos Stagnaro, Yolanda Torres, Maria Carmen Viana, Cristian Vladescu, Zahari Zarkov, Ronald C. Kessler

**Affiliations:** 1 Department of Psychology, University of Pennsylvania, Philadelphia, PA, USA; 2 San Diego State University/University of California, San Diego Joint Doctoral Program in Clinical Psychology, CA, USA; 3 Department of Psychiatry and School of Public Health, University of California San Diego, La Jolla, CA, USA; 4 VA San Diego Healthcare System, San Diego, CA, USA; 5 Department of Psychiatry & Mental Health and South African Medical Council Research Unit on Risk and Resilience in Mental Disorders, University of Cape Town, South Africa; 6 Center for Reducing Health Disparities, UC Davis Health System, Sacramento, CA, USA; 7 College of Medicine, University of Al-Qadisiya, Diwaniya governorate, Iraq; 8 Health Services Research Unit, IMIM-Hospital del Mar Medical Research Institute, Barcelona, Spain; 9 Department of Medicine and Life Sciences, Pompeu Fabra University (UPF), Barcelona, Spain; 10 Centro de Investigación Biomédica en Red de Epidemiología y Salud Pública (CIBERESP), Instituto de Salud Carlos III, Madrid, Spain; 11 Brain and Mind Institute and Medical College East Africa, the Aga Khan University, Nairobi, Kenya; 12 National Institute of Psychiatry Ramón de la Fuente Muñiz, Mexico City, Mexico; 13 Department of Psychiatry, Stony Brook University School of Medicine, Stony Brook, NY, USA; 14 Universitair Psychiatrisch Centrum – Katholieke Universiteit Leuven (UPC-KUL), Campus Gasthuisberg, Leuven, Belgium; 15 School of Psychology, Ulster University, Coleraine, UK; 16 Lisbon Institute of Global Mental Health and Chronic Diseases Research Center, NOVA Medical School, NOVA University of Lisbon, Lisbon, Portugal; 17 Survey Research Center, Institute for Social Research, University of Michigan, Ann Arbor, MI, USA; 18 IRCCS Istituto Centro San Giovanni di Dio Fatebenefratelli, Brescia, Italy; 19 Department of Developmental Psychology, University of Groningen, Groningen, The Netherlands; 20 Department of Psychiatry, University College Hospital, Ibadan, Nigeria; 21 Research, Teaching and Innovation Unit, Parc Sanitari Sant Joan de Déu, Sant Boi de Llobregat, Barcelona, Spain; 22 Centro de Investigación Biomédica en Red de Salud Mental (CIBERSAM), Instituto de Salud Carlos III, Madrid, Spain; 23 Department of Psychiatry and Clinical Psychology, St George Hospital University Medical Center, Beirut, Lebanon; 24 Institute for Development, Research, Advocacy and Applied Care (IDRAAC), Beirut, Lebanon; 25 Faculty of Applied Studies, University of Lower Silesia, Wroclaw, Poland; 26 Institut de Psychologie, EA 4057, Université Paris Cité, Paris, France; 27 Department of Health Care Policy, Harvard Medical School, Boston, MA, USA; 28 Unidad de Docencia, Investigación y Formación en Salud Mental (UDIF-SM), Gerencia Salud Mental, Servicio Murciano de Salud, Murcia, Spain; 29 Department of Mental Health, Graduate School of Medicine, The University of Tokyo, Tokyo, Japan; 30 School of Public Health and Administration, Universidad Peruana Cayetano Heredia, Lima, Peru; 31 Faculty of Social Sciences, Colegio Mayor de Cundinamarca University, Bogota, Colombia; 32 Department of Psychological Medicine, University of Otago, Dunedin, New Zealand; 33 The Matilda Centre for Research in Mental Health and Substance Use, University of Sydney, Australia; 34 Departamento de Psiquiatría y Salud Mental, Facultad de Medicina, Universidad de Buenos Aires, Argentina; 35 Center for Excellence on Research in Mental Health, CES University, Medellín, Colombia; 36 Department of Social Medicine, Postgraduate Program in Public Health, Federal University of Espírito Santo, Vitoria, Brazil; 37 National Institute of Health Services Management, Bucharest, Romania; 38 University Titu Maiorescu, Bucharest, Romania; 39 Department of Mental Health, National Center of Public Health and Analyses, Sofia, Bulgaria

**Keywords:** classification, diagnosis, epidemiology, generalized anxiety disorder, global mental health, worry

## Abstract

**Background:**

Around the world, people living in objectively difficult circumstances who experience symptoms of generalized anxiety disorder (GAD) do not qualify for a diagnosis because their worry is not ‘excessive’ relative to the context. We carried out the first large-scale, cross-national study to explore the implications of removing this excessiveness requirement.

**Methods:**

Data come from the World Health Organization World Mental Health Survey Initiative. A total of 133 614 adults from 12 surveys in Low- or Middle-Income Countries (LMICs) and 16 surveys in High-Income Countries (HICs) were assessed with the Composite International Diagnostic Interview. Non-excessive worriers meeting all other *DSM-5* criteria for GAD were compared to respondents meeting all criteria for GAD, and to respondents without GAD, on clinically-relevant correlates.

**Results:**

Removing the excessiveness requirement increases the global lifetime prevalence of GAD from 2.6% to 4.0%, with larger increases in LMICs than HICs. Non-excessive and excessive GAD cases worry about many of the same things, although non-excessive cases worry more about health/welfare of loved ones, and less about personal or non-specific concerns, than excessive cases. Non-excessive cases closely resemble excessive cases in socio-demographic characteristics, family history of GAD, and risk of temporally secondary comorbidity and suicidality. Although non-excessive cases are less severe on average, they report impairment comparable to excessive cases and often seek treatment for GAD symptoms.

**Conclusions:**

Individuals with non-excessive worry who meet all other *DSM-5* criteria for GAD are clinically significant cases. Eliminating the excessiveness requirement would lead to a more defensible GAD diagnosis.

## Introduction

The diagnostic threshold for a disorder serves a gatekeeping function (Halpin, 2016). Individuals who meet this threshold are eligible for health care, disability income, and other resources that may be unavailable to individuals who fall short of the threshold. In resource-limited systems, categorical decisions are unavoidable, making diagnosis necessary even for forms of psychopathology that are dimensional in nature (Haslam, McGrath, Viechtbauer, & Kuppens, 2020; Ruscio, 2019). Given the personal and public health impact of these decisions, where we draw the threshold matters.

Unfortunately, it is not always clear where diagnostic thresholds should be drawn. A compelling example is offered by generalized anxiety disorder (GAD). GAD is centrally defined by worry, yet worry is experienced to some degree by most people. To impose a threshold, the *Diagnostic and Statistical Manual of Mental Disorders*, fifth edition (*DSM-5*; American Psychiatric Association [APA], 2013) requires the worry to focus on multiple topics, occur most days for six months, be excessive and uncontrollable, be accompanied by three associated symptoms of anxiety, and cause clinically significant distress or impairment.

Is this threshold optimal for separating normal from pathological worry? There are indications that it is not. Considerable evidence exists that the GAD diagnosis misses individuals with clinically significant anxiety (Andrews et al., 2010). Subthreshold GAD, variably defined, is associated with a disease burden comparable to the full disorder (Haller, Cramer, Lauche, Gass, & Dobos, 2014). Subthreshold GAD is also common in clinical settings (Olivares et al., 2013), with one study reporting that 3 in 4 adults seeking treatment for clinically relevant worry fell short of a GAD diagnosis by a single diagnostic criterion (Lawrence & Brown, 2009).

A particularly problematic criterion is the requirement that worry must be ‘excessive.’ Like the requirement of ‘unrealistic’ worry, which was discarded in *DSM-IV*, there is no accepted standard for what constitutes excessive worry (Andrews et al., 2010; Starcevic, Portman, & Beck, 2012). Perhaps for this reason, clinical raters often disagree whether worry is excessive, and removing this criterion improves diagnostic reliability (Wittchen, Kessler, Zhao, & Abelson, 1995). Importantly, excessiveness is highly correlated with uncontrollability of worry (Gordon & Heimberg, 2011; Rutter & Brown, 2015), suggesting that it may be unnecessary to require both. Of the two, uncontrollability has superior incremental validity (Hallion & Ruscio, 2013) and is supported by research linking cognitive control deficits with worry (e.g. Gustavson et al., 2020; Hallion, Ruscio, & Jha, 2014; Zetsche, Bürkner, & Schulze, 2018), arguing indirectly for removing excessiveness.

Based on these considerations, Ruscio et al. (2005) investigated the impact of removing the excessiveness requirement. Analyzing an epidemiological sample of U.S. adults, they found that lifetime prevalence of *DSM-IV* GAD increased by approximately 40% when excessiveness was omitted. Although non-excessive GAD was somewhat milder than excessive GAD, non-excessive cases reported substantial persistence, comorbidity, role impairment, and treatment-seeking, supporting their inclusion in the GAD diagnosis. Subsequent studies examining a narrower range of outcomes obtained similar results (Beesdo-Baum et al., 2011; Lee, Ma, Tsang, & Kwok, 2009; Ruscio et al., 2007).

These findings, although suggestive, did not lead to the removal of excessiveness in *DSM-5*. In fact, despite pointed criticism (Shear, 2012), excessiveness was *added* as a qualifier for worry when revising the GAD definition for the 11th edition of the *International Classification of Diseases* (World Health Organization, 2019). There are likely at least two reasons for this. First, eliminating excessiveness might raise concerns about over-pathologizing, especially given the ubiquity of worry. To address these concerns, there is a need to demonstrate that broadening GAD to encompass non-excessive cases yields improvements in clinical utility that justify the increased prevalence and associated costs. As the primary stated purpose of *DSM-5* diagnoses is to facilitate clinical care (APA, 2013), it is especially important to evaluate whether non-excessive GAD is clinically significant.

Second, the impact of eliminating excessiveness has been investigated in only a few high-income countries. Given cross-national differences in GAD prevalence (Marques, Robinaugh, LeBlanc, & Hinton, 2011; Ruscio et al., 2017), there is a need to evaluate impact in other parts of the world (Lewis-Fernández et al., 2010; Stein, Scott, de Jonge, & Kessler, 2017). It is plausible that there are differing cultural standards for what is considered excessive worry. Moreover, countries vary in exposure to objectively stressful circumstances that set the stage for worry. Extreme poverty, limited health services, political instability, and other challenges faced disproportionately in lower-income countries may lead to chronic worry that is not ‘excessive’ given the context but may still be disabling and merit treatment (Kessler & Wittchen, 2002; Stein & Seedat, 2007).

We carried out the first cross-national study to explore the implications of removing excessiveness from the GAD diagnosis. We updated prior research on this topic by examining *DSM-5* GAD, which is more severe than *DSM-IV* GAD because it includes individuals whose symptoms co-occur with major mood episodes (Ruscio, Hallion, Demyttenaere, Lee, & Lim, 2018), and we expanded on prior research in several ways. First, to evaluate the clinical significance of non-excessive GAD, we considered an expanded set of outcomes of interest to clinicians. This included, for the first time, a systematic comparison of the content of excessive and non-excessive worry. Second, we supplemented inspection of correlates with formal models in which GAD meeting full *DSM-5* criteria was compared to non-excessive GAD in predicting the subsequent onset and persistence of temporally secondary outcomes. This allowed us to explore whether the prognostic value of the GAD diagnosis might be improved by capturing non-excessive cases. Third, we utilized a much larger and more diverse sample than previous studies, including respondents from 25 countries representing all regions of the globe. This increased statistical power to detect differences between excessive and non-excessive GAD, while also providing a first look at the global implications of the excessiveness requirement.

## Method

### Sample

The World Mental Health (WMH) surveys are a coordinated set of community epidemiological surveys administered to probability samples of the non-institutionalized population in countries throughout the world (https://www.hcp.med.harvard.edu/wmh/). Data for the current report came from 28 WMH surveys, 12 carried out in countries classified by the World Bank as Low- or Middle-Income (LMICs; Brazil, Bulgaria, Colombia, Iraq, Lebanon, Mexico, Peru, Romania, South Africa, Ukraine) and 16 in countries classified as High-Income (HICs; Argentina, Australia, Belgium, France, Germany, Israel, Italy, Japan, Netherlands, New Zealand, Northern Ireland, Poland, Portugal, Spain, United States). There were two surveys each in Bulgaria, Colombia, and Spain. Twenty surveys were based on nationally representative household samples, whereas one was representative of a selected region (Murcia, Spain), three of selected metropolitan areas (São Paulo, Brazil; Medellin, Colombia; 11 metropolitan areas in Japan), and four of all urbanized areas in the country (Argentina, Colombia, Mexico, Peru). Response rates ranged from 45.9% (France) to 97.2% (Medellin) and averaged 71.4% across surveys (online Supplementary Table S1).

The interview schedule was developed in English and translated into other languages using a standardized WHO translation, back-translation, expert review, and harmonization protocol (Kessler & Üstün, 2004). Interviews were administered face-to-face in respondents' homes after obtaining informed consent using procedures approved by local Institutional Review Boards. Interviews had two parts. Part I was administered to one, or in a few surveys two, randomly-selected adults in each sampled household. Part I contained assessments of core mental disorders, including GAD. A Part I weight adjusted for differential probabilities of selection within and between households. Part II, which included questions about other mental disorders and correlates, was then administered to 100% of Part I respondents who met lifetime criteria for any Part I disorder plus a probability subsample of other Part I respondents. A Part II weight equal to the inverse of the probability of selection into Part II was used to restore the representativeness of the Part II sample. A third weight was then applied to the Part II sample to calibrate discrepancies between sample and population distributions on the cross-classification of Census socio-demographic and geographic variables.

### Measures

#### Interview schedule

All surveys used the WHO Composite International Diagnostic Interview (CIDI; Kessler & Üstün, 2011), a fully-structured diagnostic interview administered by trained lay interviewers. Consistent interviewer training and quality control monitoring procedures were used across surveys (Pennell et al., 2008). In addition to GAD, 16 potentially comorbid *DSM-IV* disorders were assessed, including anxiety (panic disorder, agoraphobia, social phobia, specific phobia, posttraumatic stress disorder, separation anxiety disorder), mood (major depressive disorder, bipolar spectrum disorder), substance-related (alcohol abuse or dependence, drug abuse or dependence), and disruptive behavior disorders (attention-deficit/hyperactivity disorder, oppositional defiant disorder, conduct disorder, intermittent explosive disorder, bulimia nervosa, binge eating disorder). Diagnoses based on the CIDI have been shown to have good concordance with diagnoses based on the clinician-administered Structured Clinical Interview for *DSM-IV* (SCID; First, Spitzer, Gibbon, & Williams, 2002) in blinded clinical reappraisal interviews (Ghimire, Chardoul, Kessler, Axinn, & Adhikari, 2013; Haro et al., 2008; Kessler et al., 2020; Kimerling et al., 2014; Montoya Gonzalez et al., 2016).

Respondents who met lifetime criteria for a given disorder in the CIDI were asked about age of onset using a question series designed to encourage accurate dating (McGrath et al., 2023). Subsequent disorder-specific questions asked about course of illness, current prevalence, and treatment-seeking. In addition, separate sections considered in this report assessed socio-demographics, suicidality, and family history of GAD. A more detailed description of the CIDI is presented elsewhere (Kessler, Heeringa, Pennell, Sampson, & Zaslavsky, 2017).

#### GAD assessment

The CIDI assessment of GAD begins with retrospective questions about lifetime episodes of worrying. Respondents who report such episodes are shown a list of possible worry topics (including spaces for ‘Other’ self-nominated topics) and asked to describe the focus of their worries. Interviewers probe for up to three examples and record all topics mentioned. Respondents reporting multiple topics are then asked whether their worry or anxiety ‘was *ever* excessive or unreasonable or a lot stronger than it should have been?’ We classified respondents as having excessive or non-excessive worry based on this question. Subsequent questions were used to determine whether other GAD diagnostic criteria were met.

In the U.S. WMH survey, GAD diagnoses based on the CIDI had good concordance with diagnoses based on blinded clinical reassessments using the SCID (Ruscio et al., 2005). Clinical reappraisal studies in other WMH surveys, although not evaluating GAD in isolation, found good concordance between CIDI and SCID diagnoses for any anxiety disorder including GAD (Haro et al., 2008; Kessler et al., 2020; Montoya Gonzalez et al., 2016). Consistent with our previous work (Ruscio et al., 2017, 2018), we generated *DSM-5* GAD diagnoses by removing the *DSM-IV* hierarchical exclusion of a GAD diagnosis when symptoms occur exclusively during a mood disorder, as this was the only diagnostic difference between *DSM-IV* and *DSM-5* GAD. Age of onset of GAD was assessed using probing methods demonstrated to improve dating accuracy (Knäuper, Cannell, Schwarz, Bruce, & Kessler, 1999). Respondents with 12-month GAD were asked additional questions about functioning, including an expanded Sheehan Disability Scale (Leon, Olfson, Portera, Farber, & Sheehan, 1997) that assessed role impairment caused by GAD during the most severe month in the year before interview.

### Analysis methods

Analyses compared three mutually exclusive groups of respondents: those without GAD, those with non-excessive GAD (i.e. meeting all *DSM-5* criteria other than excessiveness), and those with excessive GAD (i.e. meeting all *DSM-5* criteria including excessiveness). We examined the data using simple cross-tabulations, regression analyses, and – in the case of models predicting the associations of lifetime GAD with subsequent first onset of temporally secondary comorbidity and suicidality – discrete-time survival models with person-year as the unit of analysis (Singer & Willett, 1993). All analyses controlled for basic socio-demographic variables. All regression analyses and survival analyses of dichotomous outcomes used modified Poisson models with robust error variances to estimate risk ratios (RRs) directly (Mittinty & Lynch, 2023). Given that WMH data are weighted and geographically clustered, the Taylor series method was used to calculate design-based standard errors of proportions and 95% confidence intervals of RRs (Wolter, 2007). All analyses were carried out using Stata Statistical Software, Release 16.1 (StataCorp LLC, 2020).

## Results

### Prevalence and persistence

Globally, broadening the diagnosis to include non-excessive cases increases CIDI GAD prevalence estimates by approximately 50% over prevalence based on full *DSM-5* criteria, with 30-day prevalence rising from 0.5% to 0.8%, 12-month prevalence rising from 1.3% to 1.9%, and lifetime prevalence rising from 2.6% to 4.0% (online Supplementary Table S2). Excessive lifetime cases are somewhat more likely to persist to the interview year (50.9%) than non-excessive cases (43.4%; χ^2^_1_ = 26.9, *p* < 0.001). However, among 12-month cases, excessive GAD is no more likely to persist throughout the year (41.1%) than non-excessive GAD (43.7%; χ^2^_1_ < 0.1, *p* = 0.977).

As hypothesized, non-excessive GAD is found disproportionately in LMICs (χ^2^_1_ = 27.9, *p* < 0.001), with non-excessive cases making up 44.3% of broadly-defined lifetime GAD in LMICs compared to 33.6% in HICs (online Supplementary Table S2). Despite this, GAD is more persistent in LMICs, with more lifetime cases persisting to the interview year in LMICs than HICs for non-excessive (52.0% *v.* 40.1%; χ^2^_1_ = 15.5, *p* < 0.001) as well as excessive (61.4% *v.* 48.4%; χ^2^_1_ = 23.8, *p* < 0.001) GAD.

### Severity

Reflecting the seriousness of their condition, fully half (50.5%) of non-excessive cases meet at least one of three indicators indexing severe GAD: *frequent* difficulty controlling worry (as opposed to occasional difficulty, which meets the diagnostic requirement), *severe* distress (as opposed to moderate distress, which meets the diagnostic requirement), or *extreme* impairment (as opposed to moderate impairment, which meets the diagnostic requirement) (online Supplementary Table S3). By comparison, two-thirds (66.1%) of excessive cases meet at least one of these indicators, and the number of indicators met is higher for excessive than non-excessive cases (χ^2^_3_ = 28.9, *p* < 0.001). To account for this difference in severity, we repeated each subsequent analysis adjusting for the number of severity indicators met (0–3). This allowed us to determine whether differences observed between non-excessive and excessive GAD were attributable to other GAD severity features.

### Worry content

Non-excessive cases worry about many of the same topics as excessive cases (Table 1). The most common worry topic is finances, reported by nearly half of non-excessive (49.4%) and excessive (47.8%) cases. The two groups also report comparable worry about family relationships and about a wide range of societal problems. However, the groups diverge in their worry about other topics. Non-excessive cases (32.5%) focus more on the health and welfare of loved ones than excessive cases (25.9%) – a group difference that is highly specific and does not extend to other concerns about one's social network. By contrast, excessive cases focus more on personal problems (excluding finances) than non-excessive cases. Excessive worry is also more likely to be diffuse in content (e.g. worrying about ‘everything’) than non-excessive worry. These differences persist after adjusting for GAD severity, suggesting that variation in worry content relates specifically to excessiveness.

**Table 1.** Worry content^a^ of non-excessive and excessive *DSM-5* GAD

	Non-excessive	Excessive	Excessive v. Non-excessive			
			Unadjusted		Adjusted	
Topic	% (s.e.)	% (s.e.)	χ^2^_1_	*p*	χ^2^_1_	*p*
Diffuse worries						
Everything	21.1 (1.2)	34.3 (1.0)	57.2	<0.001	44.2	<0.001
Nothing in particular	8.1 (0.8)	10.3 (0.6)	3.8	0.051	5.7	0.017
Any diffuse worries	28.0 (1.3)	41.4 (1.1)	52.7	<0.001	43.6	<0.001
Personal problems						
Finances	49.4 (1.5)	47.8 (1.1)	2.1	0.144	1.5	0.223
Work/school	17.7 (1.0)	26.7 (0.9)	12.8	<0.001	14.4	<0.001
Social life	12.4 (0.9)	22.5 (0.9)	30.4	<0.001	27.3	<0.001
Love life	21.5 (1.1)	30.0 (1.0)	7.3	0.007	7.1	0.008
Physical appearance	12.7 (0.8)	21.5 (0.9)	20.5	<0.001	20.1	<0.001
Health (self)^b^	36.4 (1.3)	41.6 (1.0)	10.1	0.001	6.1	0.014
Any personal problems	72.1 (1.4)	72.2 (1.0)	1.1	0.294	0.7	0.408
Network problems						
Relationships with family	36.7 (1.3)	38.2 (1.1)	0.1	0.773	0.1	0.729
Relationships at school or work	11.1 (0.8)	16.5 (0.7)	7.5	0.006	7.3	0.007
Health or welfare of loved ones	32.5 (1.4)	25.9 (1.0)	10.8	0.001	7.9	0.005
Being away from home or apart from loved ones	9.0 (0.7)	12.3 (0.7)	7.1	0.008	5.7	0.017
Any network problems	60.6 (1.3)	57.0 (1.1)	7.8	0.005	7.1	0.008
Societal problems						
Crime/violence	9.3 (0.9)	9.1 (0.6)	0.6	0.419	0.9	0.341
The economy	5.7 (0.7)	4.7 (0.4)	1.0	0.308	0.9	0.355
The environment (e.g. global warming)	3.5 (0.5)	5.0 (0.5)	2.6	0.106	2.5	0.113
Moral decline of society (e.g. commercialism)	6.1 (0.7)	7.9 (0.6)	1.9	0.163	2.3	0.133
War/revolution	5.9 (0.6)	5.8 (0.5)	0.7	0.410	1.1	0.299
Any societal problems	18.0 (1.1)	17.8 (0.8)	<0.1	0.958	<0.1	0.848
Other worry topics^c^	5.1 (0.5)	4.0 (0.4)	0.1	0.709	0.5	0.466

a Values represent the proportion of respondents with non-excessive (*n* = 2096) and excessive (*n* = 3595) lifetime GAD, respectively, who endorsed each worry topic. Within each problem domain, the final (‘any’) category reflects the proportion of respondents who endorsed at least one worry topic within that domain. All χ^2^ tests controlled for age at interview, sex, and country. Adjusted tests additionally controlled for severity, which was a 0–3 score summing across the three dichotomous indicators of uncontrollability, distress, and impairment.

b Includes worry about physical health, mental health, or substance use.

c Includes worry about any specific problem other than those listed above.

### Socio-demographic characteristics

Non-excessive and excessive GAD have very similar socio-demographic correlates (Table 2). Both are elevated among females, those previously married (divorced, separated, or widowed), and those with an ‘Other’ employment status (mainly unemployed or disabled). Both groups also have similar patterns of associations with lower educational attainment and low household income compared to non-cases, although the associations are modest in magnitude. The only significant difference between GAD groups is that excessive cases are younger on average than non-excessive cases (χ^2^_3_ = 30.6, *p* < 0.001), a difference that remains after adjusting for severity. This converges with hazard rates showing that excessive GAD typically begins at an earlier age than non-excessive GAD (online Supplementary Fig. S1). Whereas excessive GAD most often begins in adolescence or early adulthood, non-excessive GAD has a relatively consistent risk of onset across the lifespan.

**Table 2.** Socio-demographic correlates^a^ of non-excessive and excessive *DSM-5* GAD

			Excessive v. non-excessive	
	Non-excessive	Excessive	Unadjusted	Adjusted
Predictors	RR (95% CI)	RR (95% CI)	RR (95% CI)	RR (95% CI)
Age (years)^b^				
18–29	0.4* (0.3–0.5)	1.3* (1.1–1.5)	1.5* (1.3–1.6)	1.4* (1.3–1.6)
30–44	0.9 (0.8–1.1)	1.8* (1.6–2.0)	1.3* (1.2–1.4)	1.2* (1.1–1.3)
45–59	1.2* (1.0–1.4)	1.8* (1.6–2.1)	1.2* (1.1–1.3)	1.2* (1.1–1.3)
60+	1.0 (1.0–1.0)	1.0 (1.0–1.0)	1.0 (1.0–1.0)	1.0 (1.0–1.0)
χ^2^_3_ (*p*)	37.8 (<0.001)	41.4 (<0.001)	30.6 (<0.001)	28.4 (<0.001)
Sex^b^				
Female	1.8* (1.6–2.1)	1.8* (1.7–2.0)	1.0 (0.9–1.0)	1.0 (0.9–1.0)
Male	1.0 (1.0–1.0)	1.0 (1.0–1.0)	1.0 (1.0–1.0)	1.0 (1.0–1.0)
χ^2^_1_ (*p*)	106.1 (<0.001)	188.2 (<0.001)	0.2 (0.645)	0.2 (0.622)
Marital status^b^				
Never married	0.7* (0.6–0.8)	0.9 (0.8–1.0)	1.1* (1.0–1.1)	1.1 (1.0–1.1)
Divorced/separated/widowed	1.7* (1.4–1.9)	1.6* (1.4–1.8)	1.0 (0.9–1.0)	1.0 (0.9–1.0)
Currently married	1.0 (1.0–1.0)	1.0 (1.0–1.0)	1.0 (1.0–1.0)	1.0 (1.0–1.0)
χ^2^_2_ (*p*)	36.7 (<0.001)	40.6 (<0.001)	2.7 (0.067)	2.7 (0.061)
Employment status^c^				
Student	0.7 (0.4–1.0)	1.0 (0.8–1.4)	1.1 (1.0–1.2)	1.1 (1.0–1.2)
Homemaker	1.1 (0.9–1.3)	1.2 (1.0–1.4)	1.0 (0.9–1.1)	1.0 (0.9–1.1)
Retired	0.7* (0.6–0.9)	0.6* (0.5–0.7)	0.9 (0.8–1.1)	1.0 (0.9–1.1)
Other	1.6* (1.3–1.9)	1.7* (1.5–2.0)	1.0 (1.0–1.1)	1.0 (0.9–1.1)
Employed	1.0 (1.0–1.0)	1.0 (1.0–1.0)	1.0 (1.0–1.0)	1.0 (1.0–1.0)
χ^2^_4_ (*p*)	9.8 (<0.001)	24.4 (<0.001)	1.3 (0.258)	0.5 (0.702)
Educational attainment^c^				
Low	1.2 (1.0–1.4)	1.0 (0.8–1.1)	0.9 (0.9–1.0)	0.9 (0.9–1.0)
Low-average	1.2* (1.0–1.4)	1.1* (1.0–1.3)	1.0 (0.9–1.1)	1.0 (0.9–1.0)
High-average	1.1 (0.9–1.3)	1.0 (0.9–1.1)	1.0 (0.9–1.0)	1.0 (0.9–1.0)
High	1.0 (1.0–1.0)	1.0 (1.0–1.0)	1.0 (1.0–1.0)	1.0 (1.0–1.0)
χ^2^_3_ (*p*)	1.7 (0.162)	2.9 (0.033)	1.0 (0.377)	1.2 (0.316)
Household income^c^				
Low	1.2* (1.0–1.4)	1.2* (1.1–1.4)	1.0 (0.9–1.1)	1.0 (0.9–1.1)
Low-average	1.1 (0.9–1.4)	0.9 (0.8–1.1)	0.9 (0.9–1.0)	0.9 (0.8–1.0)
High-average	1.1 (0.9–1.3)	1.1 (1.0–1.2)	1.0 (0.9–1.1)	1.0 (0.9–1.1)
High	1.0 (1.0–1.0)	1.0 (1.0–1.0)	1.0 (1.0–1.0)	1.0 (1.0–1.0)
χ^2^_3_ (*p*)	1.7 (0.167)	7.0 (<0.001)	1.3 (0.272)	1.5 (0.201)

a The risk ratios (RRs) were estimated in modified Poisson regression models, with each sociodemographic variable predicting each GAD group. All models controlled for age at interview, sex, and country. Adjusted models additionally controlled for severity, which was a 0–3 score summing across the three dichotomous indicators of uncontrollability, distress, and impairment.

b Analyses were performed in the Part I sample. Mutually exclusive groups with non-excessive (*n* = 2096) and excessive (*n* = 3595) GAD were each compared with respondents having no lifetime GAD (*n* = 127 923), and then compared with each other.

c Analyses were performed in the Part II sample, which included 1983 respondents with non-excessive GAD, 3418 respondents with excessive GAD, and 70 749 respondents with no lifetime GAD.

* Significant at the 0.05 level.

### Family history of GAD

The likelihood of having a parent with GAD is much higher for non-excessive (5.2%; RR = 2.5) and excessive (9.7%; RR = 3.6) cases than for individuals without GAD (1.9%; both χ^2^_1_ > 24.3, both *p* < 0.001). The difference between excessive and non-excessive cases is also significant, but far smaller (RR = 1.2; χ^2^_1_ = 10.2, *p* = 0.002). Similarly, excessive cases have more close relatives who are very worried or anxious compared to non-excessive cases (χ^2^_2_ = 5.6, *p* = 0.004), but the difference is small and significant only for the risk of having two or more anxious relatives (RR = 1.1). All group differences hold when adjusting for severity, suggesting that excessive GAD is modestly more familial than non-excessive GAD.

### Temporally secondary comorbidity

The vast majority of respondents with a lifetime history of non-excessive (73.7%) and excessive (87.1%) GAD had at least one other lifetime mental disorder occur subsequent to the onset of GAD, compared to just 28.2% of respondents without GAD (online Supplementary Table S4). Strikingly, individuals with non-excessive GAD are more than eight times as likely as those without GAD to develop a later mental disorder (Table 3). While there are some disorders for which excessive GAD is a stronger predictor than non-excessive GAD, the two GAD groups do not differ overall in their risk of subsequent psychopathology (RR = 1.2, χ^2^_1_ = 3.0, *p* = 0.081). GAD also predicts the course of subsequent disorders, operationalized as 12-month persistence among lifetime cases (online Supplementary Table S5). There is no difference in this regard between excessive and non-excessive GAD (RR = 1.0; χ^2^_1_ < 0.1, *p* = 0.989): Both are associated with a 30% increase in risk of experiencing a persistent comorbid disorder.

**Table 3.** Associations^a^ of non-excessive and excessive *DSM-5* GAD with the onset of subsequent *DSM-IV* disorders

			Excessive v. non-excessive	
	Non-excessive	Excessive	Unadjusted	Adjusted
*DSM-IV* disorder	RR (95% CI)	RR (95% CI)	RR (95% CI)	RR (95% CI)
Anxiety disorder				
Panic disorder^b^	6.4* (4.9–8.4)	11.7* (9.5–14.2)	1.8* (1.3–2.5)	1.7* (1.2–2.4)
Agoraphobia^b^	3.1* (1.8–5.3)	8.3* (6.4–10.7)	2.7* (1.5–4.7)	2.3* (1.3–4.1)
Social phobia^b^^,^^c^	2.8* (1.9–4.2)	12.1* (10.3–14.3)	4.3* (2.8–6.7)	4.0* (2.6–6.2)
Specific phobia^b^^,^^d^	3.4* (2.3–5.1)	4.1* (3.2–5.2)	1.2 (0.8–1.9)	1.2 (0.8–1.9)
Posttraumatic stress disorder^e^	9.2* (7.5–11.2)	7.8* (6.7–9.1)	0.9 (0.7–1.1)	0.8 (0.6–1.0)
Separation anxiety disorder^e^^,^^f^	5.1* (3.6–7.3)	6.2* (5.0–7.6)	1.2 (0.8–1.8)	1.2 (0.8–1.7)
Any anxiety disorder^e^	8.5* (7.1–10.2)	10.5* (9.1–12.1)	1.2 (1.0–1.5)	1.1 (0.9–1.4)
Mood disorder				
Major depressive disorder^b^	9.7* (8.4–11.1)	9.7* (8.7–10.8)	1.0 (0.9–1.2)	0.9 (0.8–1.1)
Bipolar spectrum disorder^b^^,^^g^	5.3* (3.9–7.2)	10.1* (8.2–12.4)	1.9* (1.3–2.7)	1.8* (1.2–2.5)
Any mood disorder^e^	10.1* (8.8–11.6)	12.1* (11.0–13.3)	1.2* (1.0–1.4)	1.1 (0.9–1.3)
Substance use disorder				
Alcohol abuse or dependence^e^	2.6* (2.1–3.3)	3.3* (2.8–3.9)	1.3 (1.0–1.7)	1.2 (0.9–1.6)
Drug abuse or dependence^e^^,^^h^	3.3* (2.3–4.7)	5.5* (4.5–6.7)	1.7* (1.1–2.4)	1.6* (1.1–2.4)
Any substance use disorder^e^	2.7* (2.2–3.4)	3.6* (3.1–4.2)	1.3* (1.0–1.7)	1.3 (1.0–1.7)
Disruptive-behavior disorder				
Attention-deficit/hyperactivity disorder^e^^,^^i^^,^^j^	4.6* (1.4–15.6)	3.5* (1.5–8.1)	0.8 (0.2–3.3)	0.5 (0.1–2.4)
Oppositional defiant disorder^e^^,^^j^^,^^k^	2.8 (0.8–9.8)	4.4* (2.6–7.4)	1.6 (0.4–6.1)	1.5 (0.4–6.3)
Conduct disorder^e^^,^^j^^,^^l^	2.1 (0.4–9.5)	3.2* (1.8–5.6)	1.5 (0.3–7.8)	1.5 (0.3–8.0)
Intermittent explosive disorder^b^^,^^m^	4.9* (2.2–11.2)	7.4* (5.5–10.0)	1.5 (0.6–3.5)	1.3 (0.5–3.0)
Bulimia nervosa^e^^,^^n^	1.8 (0.8–4.2)	6.2* (4.3–9.0)	3.4* (1.4–8.2)	3.2* (1.3–7.5)
Binge eating disorder^e^^,^^n^	3.1* (1.9–4.8)	5.4* (4.3–6.8)	1.8* (1.1–2.9)	1.8* (1.1–3.0)
Any disruptive behavior disorder^e^	4.5* (2.7–7.3)	6.1* (5.0–7.4)	1.4 (0.8–2.3)	1.3 (0.8–2.1)
Any comorbid disorder^e^	8.4* (7.1–9.9)	10.0* (8.8–11.3)	1.2 (1.0–1.5)	1.1 (0.9–1.3)

a The risk ratios (RRs) come from discrete-time survival analysis, with person-year as the unit of analysis. Each model estimated the association between variably-defined GAD and the subsequent onset of a temporally secondary disorder, controlling for age at interview, age-squared, sex, and country. Adjusted models additionally controlled for severity, which was a 0–3 score summing across the three dichotomous indicators of uncontrollability, distress, and impairment. The models for aggregate disorder categories (‘any’ disorder) based associations on the earliest-occurring disorder within the category.

b Analyses were performed in the Part I sample. Mutually exclusive groups with non-excessive (*n* = 2096) and excessive (*n* = 3595) GAD were each compared with respondents having no lifetime GAD (*n* = 127 923), and then compared with each other.

c Not assessed in Israel.

d Not assessed in Australia, Israel, South Africa, or Ukraine.

e Analyses were performed in the Part II sample, which included 1983 respondents with non-excessive GAD, 3418 respondents with excessive GAD, and 70 749 respondents with no lifetime GAD. The Part II sample for childhood disorders included 845 respondents with non-excessive GAD, 1823 respondents with excessive GAD, and 38 776 respondents with no lifetime GAD.

f Not assessed in Australia, Israel, Japan, New Zealand, Poland, South Africa, or Ukraine.

g Includes bipolar I disorder, bipolar II disorder, or subthreshold bipolar disorder as defined by Merikangas et al. (2007). Not assessed in Belgium, France, Germany, Italy, Netherlands, South Africa, Spain, or Ukraine.

h Not assessed in Portugal.

i Not assessed in Australia, Bulgaria, Israel, Japan, New Zealand, South Africa, or Ukraine.

j Recall of childhood disorders was restricted to respondents < 45 years of age at interview (with the exception of Ukraine, which was restricted to age < 40 at interview).

k Not assessed in Australia, Bulgaria, Bulgaria 2, Israel, Japan, Lebanon, New Zealand, South Africa, or Ukraine.

l Not assessed in Australia, Israel, Japan, Lebanon, New Zealand, South Africa, or Ukraine.

m Not assessed in Australia, Belgium, Bulgaria 2, France, Germany, Israel, Italy, Medellin, Mexico, Murcia, Netherlands, New Zealand, or Spain.

n Eating disorders were assessed in a random 50% of the Part II sample. Not assessed in Australia, Bulgaria, Bulgaria 2, Israel, Japan, Lebanon, Romania, South Africa, or Ukraine.

* Significant at the 0.05 level.

### Subsequent suicidality

Compared to those without GAD, individuals with non-excessive GAD are six times as likely to subsequently develop suicidal ideation, two times as likely to make a suicide plan, and nearly two times as likely to make a suicide attempt (Table 4). Excessive and non-excessive GAD differ only in their prediction of suicidal ideation (χ^2^_1_ = 10.0, *p* = 0.002); the predictive advantage for excessive GAD is modest (RR = 1.3) and disappears when controlling for severity (RR = 1.1, χ^2^_1_ = 2.0, *p* = 0.159). GAD also predicts the persistence of suicidal ideation once it develops (online Supplementary Table S6), with no difference in prediction by excessiveness (RR = 1.1; χ^2^_1_ = 0.7, *p* = 0.397).

**Table 4.** Associations^a^ of non-excessive and excessive *DSM-5* GAD with the onset of subsequent suicide-related outcomes

			Excessive v. Non-excessive	
	Non-Excessive	Excessive	Unadjusted	Adjusted
Outcome	RR (95% CI)	RR (95% CI)	RR (95% CI)	RR (95% CI)
Suicidal ideation	6.2* (5.4–7.1)	7.9* (7.3–8.7)	1.3* (1.1–1.5)	1.1 (1.0–1.3)
Suicide plan	2.1* (1.7–2.7)	2.6* (2.2–3.0)	1.2 (0.9–1.6)	1.1 (0.8–1.4)
Suicide attempt	1.8* (1.4–2.4)	1.6* (1.3–1.9)	0.9 (0.6–1.2)	0.9 (0.6–1.2)

a The risk ratios (RRs) come from discrete-time survival analysis with person-year as the unit of analysis. Analyses were performed in the Part II sample, which included 1983 respondents with non-excessive GAD, 3418 respondents with excessive GAD, and 70 749 respondents with no lifetime GAD. Each model estimated the association between variably-defined GAD and the subsequent onset of a temporally secondary suicide-related outcome. The models for suicide plan were estimated among respondents with lifetime suicidal ideation. The models for suicide attempt were estimated among respondents with lifetime suicidal ideation and also controlled for person-year lifetime suicide plan status. All models controlled for age at interview, age-squared, sex, and country. Adjusted models additionally controlled for severity, which was a 0–3 score summing across the three dichotomous indicators of uncontrollability, distress, and impairment.

* Significant at the 0.05 level.

### Treatment-seeking for GAD

Nearly half (43.4%) of non-excessive cases seek treatment specifically for GAD in their lifetime (online Supplementary Table S7). Treatment-seeking is somewhat higher among excessive cases (51.6%; χ^2^_1_ = 6.8, *p* = 0.010), but this difference declines to non-significance after adjusting for severity (χ^2^_1_ = 2.7, *p* = 0.101). In the year before interview, 18.9% of non-excessive and 26.9% of excessive 12-month cases received treatment for GAD symptoms (χ^2^_1_ = 19.6, *p* < 0.001). This group difference is attenuated, though not eliminated, after adjusting for severity (χ^2^_1_ = 12.6, *p* < 0.001).

### Impairment due to GAD

Excessive and non-excessive 12-month cases report similar functional impairment due to GAD in the past year (Table 5). Approximately 65–75% of respondents in both groups report some impairment, and approximately 50–60% report moderate or severe impairment, in at least one of the four domains assessed. Only 1 of the 12 nested responses distinguishes the two groups; this difference disappears after adjusting for GAD severity. Remarkably, non-excessive 12-month cases report an average of 30 days (s.d. = 79.6) in the past year when they were totally unable to work or carry out their normal activities because of GAD. This is comparable to the average of 33 days (s.d. = 90.9) reported by excessive 12-month cases, whether analyzed across the entire year (χ^2^_1_ = 1.5, *p* = 0.213) or as a fraction of the time spent in GAD episodes during the year (χ^2^_1_ = 2.6, *p* = 0.109).

**Table 5.** Severity of role impairment^a^ associated with 12-month non-excessive and excessive GAD

			Excessive v. Non-excessive			
	Non-excessive	Excessive	Unadjusted		Adjusted	
Variable	% (s.e.)	% (s.e.)	χ^2^_1_	*p*	χ^2^_1_	*p*
Home management						
Severe	23.1 (1.8)	25.9 (1.2)	1.5	0.218	0.1	0.745
Moderate or severe	50.0 (2.0)	52.1 (1.4)	1.2	0.277	0.1	0.721
Any impairment	67.3 (2.0)	71.0 (1.3)	3.7	0.054	2.7	0.100
Work						
Severe	25.1 (1.9)	29.0 (1.3)	0.6	0.436	<0.1	0.900
Moderate or severe	49.4 (2.0)	51.1 (1.4)	<0.1	0.851	0.9	0.345
Any impairment	64.6 (2.1)	68.7 (1.3)	0.7	0.397	0.3	0.605
Relationships						
Severe	23.1 (1.8)	30.8 (1.2)	4.8	0.028	2.0	0.157
Moderate or severe	51.1 (2.2)	58.0 (1.3)	1.0	0.318	0.1	0.713
Any impairment	69.6 (2.0)	75.0 (1.2)	2.9	0.091	2.2	0.135
Social life						
Severe	26.9 (2.0)	33.4 (1.3)	2.3	0.128	0.5	0.461
Moderate or severe	52.4 (2.2)	59.0 (1.4)	1.1	0.287	0.2	0.612
Any impairment	68.4 (2.1)	74.3 (1.2)	3.2	0.073	2.7	0.102

a Values represent the proportion of respondents with non-excessive (*n* = 935) and excessive (*n* = 1822) 12-month GAD, respectively, reporting severe (score of 7–10), severe or moderate (score of 4–10), or any (score of 1–10) impairment in each of the four Sheehan Disability Scale domains of functioning. All χ^2^ tests controlled for age at interview, sex, and country. Adjusted tests additionally controlled for severity, which was a 0–3 score summing across the three dichotomous indicators of uncontrollability, distress, and impairment.

## Discussion

This study undertook the first cross-national test of the GAD excessiveness requirement. We found that removing this requirement increases the prevalence of *DSM-5* GAD by about 50% worldwide. While the increase in prevalence may raise concerns about pathologizing normal anxiety or diagnosing the so-called ‘worried well,’ our data do not bear out these concerns. Instead, they show that non-excessive worriers who meet all other GAD criteria are very similar to excessive worriers with GAD, and very different from persons without GAD, on a wide array of clinically significant features. Where differences emerge between non-excessive and excessive GAD, they are modest in magnitude, and many disappear when other disorder features are controlled. Most important, on almost any metric of seriousness (e.g. help-seeking, functional impairment), non-excessive GAD appears to be clinically significant.

Removing excessiveness would bring GAD in line with other disorders that often emerge in the context of significant stressors. There is no requirement, for example, that sadness must be excessive for major depression to be diagnosed. Instead, major depression is distinguished from normal sadness through other criteria specifying intensity, frequency, and duration of symptoms that are judged so substantial and impairing that they warrant clinical attention. At the same time, removing excessiveness could open GAD up to criticism, previously levied at depression, that it is inappropriate to diagnose symptoms that are severe yet proportional to their context (Horwitz, 2015). Our results inform this larger debate by showing that non-excessive GAD is highly familial, persistent, disabling, and predictive of a wide range of outcomes that matter for clinical care. Whether it is appropriate to diagnose proportional yet disabling reactions to stress remains an important question for the field to resolve (Stein, Palk, & Kendler, 2021), but there seems no reason why the answer should be different for GAD than for other disorders.

Our finding that non-excessive GAD is similar to but less severe than excessive GAD is consistent with mounting evidence for the dimensional nature of anxiety (Haslam, Holland, & Kuppens, 2012; Ruscio, 2019). Given this dimensionality, any reduction in diagnostic requirements will result in new cases of lower severity, just as any increase in diagnostic criteria beyond those currently required will result in fewer cases of greater severity. The open question is where along this dimension the diagnostic threshold should be drawn for categorical decision making. Following *DSM*'s own directive that the threshold be placed at the point of clinical significance (APA, 2013), our results suggest that the *DSM-5* threshold for GAD is set too high. Non-excessive cases are just as disabled by their symptoms as excessive cases, report losing a full month in the past year due to those symptoms, and often seek treatment for those symptoms.

Why might individuals whose symptoms are so severe that they otherwise qualify for a GAD diagnosis regard their worry as non-excessive? We found that non-excessive worriers often focus on topics that might be perceived as normative (e.g. finances, well-being of loved ones), and focus less on non-specific concerns (e.g. ‘everything’) that may be easier to recognize as excessive. This may reduce insight, perhaps especially among lifelong worriers who have a limited basis for comparison. Alternatively, non-excessive worry might be a justifiable reaction to genuine deprivation or stress. We observed higher rates of non-excessive GAD in LMICs than HICs, perhaps reflecting heightened stress and uncertainty in parts of the world where caring for oneself and one's family can be a daily struggle. Greater persistence of symptoms in LMICs may also reflect a greater likelihood of ongoing stressors in those countries. Critically, an unintended consequence of the excessiveness requirement may be that individuals with low socioeconomic status or stigmatized identities – whose worries may be viewed as an understandable reaction to the stressors they face – may be less likely to receive a GAD diagnosis and gain access to quality treatment than more-advantaged patients with the same presenting complaint. We did not find a stronger association of household income with non-excessive than excessive GAD, but were unable to test whether other forms of disadvantage previously linked to chronic stress were concentrated among non-excessive cases (Hatzenbuehler, 2009; Zvolensky, Garey, & Bakhshaie, 2017). Such tests are needed to explore whether eliminating excessiveness reduces disparities in GAD diagnosis and treatment.

Our results imply that clinicians should not dismiss symptoms for being consistent with (worrisome) life circumstances, as intervention offers opportunities for symptom relief and perhaps even prevention of subsequent comorbidity and suicidality. Notably, nearly half of non-excessive cases seek help for GAD symptoms, though whether clinicians recognize their difficulties as GAD is unclear. Diagnosing these cases with an ‘other specified’ anxiety disorder is an imperfect solution, as this does not promote the use of treatments that are empirically supported for GAD. Removing the excessiveness requirement may also have implications for early intervention. Recently, there has been growing attention to anxiety screening in primary healthcare, with the U.S. Preventive Services Task Force (2023) recommending that all adults under age 65 be screened for anxiety. As GAD is the most prevalent anxiety disorder in primary care (DeMartini, Patel, & Fancher, 2019; Lieb, Becker, & Altamura, 2005) and is the focus of recommended screening tools (U.S. Preventive Services Task Force, 2023), it is likely to be among the most common anxiety disorders detected through screening. Knowing where to set the cutoff for GAD is crucial, as it will determine who will be referred for further services, which will have direct implications for access and cost.

### Limitations and future directions

Our study had several limitations. First, we asked respondents directly about excessiveness. *DSM-5* does not specify who should decide whether worry is excessive; we prioritized self-reports, but other reporters may have answered differently. Second, we were unable to explore respondents' understanding of ‘excessive’ worry. Given ambiguity around the meaning of excessiveness (Shear, 2012), there is a need to better understand what factors people consider when appraising their worry as excessive. Third, we did not probe for culturally specific worry topics (Hinton, Hsia, Park, Rasmussen, & Pollack, 2009). However, past research suggests that GAD worries are more similar than dissimilar across cultures (Marques et al., 2011), and our interview offered flexibility by using broad categories and by recording up to three ‘Other’ topics beyond the standard list. Fourth, we tested excessiveness as a predictor of subsequent outcomes, but the temporal order of predictors and outcomes was determined via retrospective reports. Prospective longitudinal studies are needed to replicate these results.

Our analyses did not establish whether these results are unique to excessiveness or whether similar results might be observed for other contested features of GAD (Andrews et al., 2010). Future studies could use the analytic framework employed here to evaluate the population-level impact of revising other diagnostic criteria. All criteria that require revision could then be studied together (cf. Beesdo-Baum et al., 2011; Ruscio et al., 2007) to determine which combination optimizes the distinction between clinically significant GAD and normal-range anxiety. Finally, we focused on clinically important outcomes that any useful diagnosis should predict. Future work should test an equally wide range of risk factors, to ensure that removing excessiveness preserves the validity of the diagnosis as well as its clinical utility. The main risk factor we considered, family history of GAD, was heightened in excessive compared to non-excessive cases, although the difference was small. Other risk factors, such as life stress, might plausibly show the opposite pattern, given the more widely distributed risk of onset over the lifespan for non-excessive than excessive GAD.

## Conclusions

Individuals who deny that their worry is excessive – but meet all other GAD criteria – experience nontrivial mental health symptoms associated with substantial functional impairment, help-seeking, and risk of adverse mental health outcomes. Although somewhat milder in severity than excessive GAD cases, non-excessive cases are affected in ways that unambiguously meet the *DSM-5* requirement of a ‘clinically significant disturbance’ that is ‘associated with significant distress or disability’ (APA, 2013, p. 20). Lowering the GAD threshold to include these worriers would improve clinical coverage, promote recognition of symptoms that are important for effective prognosis and clinical management, and yield a more defensible GAD diagnosis.

## Supporting information

Ruscio et al. supplementary material Ruscio et al. supplementary material

## Supplementary material

The supplementary material for this article can be found at https://doi.org/10.1017/S003329172400182X
