# Supplementary material for: The case for eliminating excessive worry as a requirement for generalized anxiety disorder: a cross-national investigation
Source: Psychol Med. 2024 Oct 4;54(12):3447–58. doi: 10.1017/S003329172400182X (PMC11496212; doi:10.1017/S003329172400182X)
Supplement: Ruscio et al. supplementary material [file S003329172400182Xsup001.docx]

**Online Supplement**

*Ruscio et al.*

*The case for eliminating excessive worry as a requirement for generalized anxiety disorder: A cross-national investigation*

| **Table S1. WMH sample characteristics by World Bank income categories*^a^*** | | | | | | | | |
| --- | --- | --- | --- | --- | --- | --- | --- | --- |
|  | | | | | | | | |
|  |  |  |  |  | **Sample size** | | |  |
| **Country** | **Survey*^b^*** | **Sample characteristics*^c^*** | **Field dates** | **Age range** | **Part I** | **Part II** | **Part II and age ≤ 44*^d^*** | **Response rate*^e^*** |
| **I. Low/middle income** | | |  |  |  |  |  |  |
| Brazil – São Paulo | São Paulo Megacity | São Paulo metropolitan area. | 2005-8 | 18-93 | 5,037 | 2,942 | 1,824 | 81.3 |
| Bulgaria | NSHS | Nationally representative. | 2002-6 | 18-98 | 5,318 | 2,233 | 741 | 72 |
| Bulgaria 2 | NSHS – 2 | Nationally representative. | 2016-17 | 18-91 | 1,508 | 578 | 202 | 61 |
| Colombia | NSMH | All urban areas of the country (approximately 73% of the total national population). | 2003 | 18-65 | 4,426 | 2,381 | 1,731 | 87.7 |
| Colombia – Medellin | MMHHS | Medellin metropolitan area | 2011-12 | 19-65 | 3,261 | 1,673 | 970 | 97.2 |
| Iraq | IMHS | Nationally representative. | 2006-7 | 18-96 | 4,332 | 4,332 | 3,227 | 95.2 |
| Lebanon | LEBANON | Nationally representative. | 2002-3 | 18-94 | 2,857 | 1,031 | 595 | 70 |
| Mexico | M-NCS | All urban areas of the country (approximately 75% of the total national population). | 2001-2 | 18-65 | 5,782 | 2,362 | 1,736 | 76.6 |
| Peru | EMSMP | Five urban areas of the country (approximately 38% of the total national population). | 2004-5 | 18-65 | 3,930 | 1,801 | 1,287 | 90.2 |
| Romania | RMHS | Nationally representative. | 2005-6 | 18-96 | 2,357 | 2,357 | 940 | 70.9 |
| South Africa^f^ | SASH | Nationally representative. | 2002-4 | 18-92 | 4,315 | 4,315 | 3,094 | 87.1 |
| Ukraine | CMDPSD | Nationally representative. | 2002 | 18-91 | 4,725 | 1,720 | 541 | 78.3 |
| **TOTAL** |  |  |  |  | 47,848 | 27,725 | 16,888 | 81.8 |
| **II. High income** | | |  |  |  |  |  |  |
| Argentina | AMHES | Eight largest urban areas of the country (approximately 50% of the total national population) | 2015 | 18-98 | 3,927 | 2,116 | 1,123 | 77.3 |
| Australia^f^ | NSMHWB | Nationally representative. | 2007 | 18-85 | 8,463 | 8,463 | 4,021 | 60 |
| Belgium | ESEMeD | Nationally representative. The sample was selected from a national register of Belgium residents. | 2001-2 | 18-95 | 2,419 | 1,043 | 486 | 50.6 |
| France | ESEMeD | Nationally representative. The sample was selected from a national list of households with listed telephone numbers. | 2001-2 | 18-97 | 2,894 | 1,436 | 727 | 45.9 |
| Germany | ESEMeD | Nationally representative. | 2002-3 | 19-95 | 3,555 | 1,323 | 621 | 57.8 |
| Israel | NHS | Nationally representative. | 2003-4 | 21-98 | 4,859 | 4,859 | 2,502 | 72.6 |
| Italy | ESEMeD | Nationally representative. The sample was selected from municipality resident registries. | 2001-2 | 18-100 | 4,712 | 1,779 | 853 | 71.3 |
| Japan | WMHJ 2002-2006 | Eleven metropolitan areas. | 2002-6 | 20-98 | 4,129 | 1,682 | 547 | 55.1 |
| Netherlands | ESEMeD | Nationally representative. The sample was selected from municipal postal registries. | 2002-3 | 18-95 | 2,372 | 1,094 | 516 | 56.4 |
| New Zealand^f^ | NZMHS | Nationally representative. | 2004-5 | 18-98 | 12,790 | 7,312 | 4,119 | 73.3 |
| Northern Ireland | NISHS | Nationally representative. | 2005-8 | 18-97 | 4,340 | 1,986 | 907 | 68.4 |
| Poland | EZOP | Nationally representative | 2010-11 | 18-65 | 10,081 | 4,000 | 2,276 | 50.4 |
| Portugal | NMHS | Nationally representative. | 2008-9 | 18-81 | 3,849 | 2,060 | 1,070 | 57.3 |
| Spain | ESEMeD | Nationally representative. | 2001-2 | 18-98 | 5,473 | 2,121 | 960 | 78.6 |
| Spain-Murcia | PEGASUS- Murcia | Murcia region. Regionally representative. | 2010-12 | 18-96 | 2,621 | 1,459 | 631 | 67.4 |
| United States | NCS-R | Nationally representative. | 2001-3 | 18-99 | 9,282 | 5,692 | 3197 | 70.9 |
| **TOTAL** |  |  |  |  | 85,766 | 48,425 | 24,556 | 64.7 |
|  |  |  |  |  |  |  |  |  |
| **III. TOTAL SAMPLE** |  |  |  |  | 133,614 | 76,150 | 41,444 | 71.4 |
|  | | | | | | | | |

^a^ The World Bank (2012) Data. Accessed May 12, 2012 at: <http://data.worldbank.org/country>. Some of the World Mental Health (WMH) countries have moved into new income categories since the surveys were conducted. The income groupings above reflect the status of each country at the time of data collection. The current income category of each country is available at the preceding URL.

^b^ NSHS (Bulgaria National Survey of Health and Stress); NSMH (The Colombian National Study of Mental Health); MMHHS (Medellín Mental Health Household Study); IMHS (Iraq Mental Health Survey); LEBANON (Lebanese Evaluation of the Burden of Ailments and Needs of the Nation); M-NCS (The Mexico National Comorbidity Survey); EMSMP (La Encuesta Mundial de Salud Mental en el Peru); RMHS (Romania Mental Health Survey); SASH (South Africa Health Survey); CMDPSD (Comorbid Mental Disorders during Periods of Social Disruption); AMHES (Argentina Mental Health Epidemiologic Survey); NSMHWB (National Survey of Mental Health and Wellbeing); ESEMeD (The European Study Of The Epidemiology Of Mental Disorders); WMHJ2002-2006 (World Mental Health Japan Survey); NZMHS (New Zealand Mental Health Survey); NISHS (Northern Ireland Study of Health and Stress); EZOP (Epidemiology of Mental Disorders and Access to Care Survey); NMHS (Portugal National Mental Health Survey); PEGASUS-Murcia (Psychiatric Enquiry to General Population in Southeast Spain-Murcia); NCS-R (The US National Comorbidity Survey Replication).

^c^ Most WMH surveys are based on stratified multistage clustered area probability household samples in which samples of areas equivalent to counties or municipalities in the US were selected in the first stage followed by one or more subsequent stages of geographic sampling (e.g., towns within counties, blocks within towns, households within blocks) to arrive at a sample of households, in each of which a listing of household members was created and one or two people were selected from this listing to be interviewed. No substitution was allowed when the originally sampled household resident could not be interviewed. These household samples were selected from Census area data in all countries other than France (where telephone directories were used to select households) and the Netherlands (where postal registries were used to select households). Several WMH surveys (Belgium, Germany, Italy, Poland, Spain-Murcia) used municipal, country resident or universal health-care registries to select respondents without listing households. The Japanese sample is the only totally un-clustered sample, with households randomly selected in each of the 11 metropolitan areas and one random respondent selected in each sample household. 20 of the 28 surveys are based on nationally representative household samples.

^d^ Recall of childhood disorders was restricted to respondents < 45 years of age at interview (with the exception of Ukraine, which was restricted to age < 40 at interview).

^e^ The response rate is calculated as the ratio of the number of households in which an interview was completed to the number of households originally sampled, excluding from the denominator households known not to be eligible either because of being vacant at the time of initial contact or because the residents were unable to speak the designated languages of the survey. The weighted average response rate is 71.4%.

^f^ For the purposes of cross-national comparisons we limit the sample to those ages 18+.

| **Table S2. Prevalence and persistence^a^ of non-excessive and excessive *DSM-5* GAD as a function of country income level** | | | | | | | | | | | | | | | | | | | | | | | |
| --- | --- | --- | --- | --- | --- | --- | --- | --- | --- | --- | --- | --- | --- | --- | --- | --- | --- | --- | --- | --- | --- | --- | --- |
|  | | | | | | | | | | | | | | | | | | | | | | | |
| **I. Lifetime, 12-month, and 30-day prevalence** | | | | | | | | | | | | | | | | | | | | | | | |
|  | **Non-excessive** | | | | | | | | | | |  | **Excessive** | | | | | | | | | | |
|  | **Lifetime** | | |  | **12-month** | | |  | **30-day** | | |  | **Lifetime** | | |  | **12-month** | | |  | **30-day** | | |
| **Country** | ***n*** | **%** | **(*SE*)** |  | ***n*** | **%** | **(*SE*)** |  | ***n*** | **%** | **(*SE*)** |  | ***n*** | **%** | **(*SE*)** |  | ***n*** | **%** | **(*SE*)** |  | ***n*** | **%** | **(*SE*)** |
| Low/middle Income | 541 | 1.1 | (0.1) |  | 289 | 0.6 | (0.0) |  | 157 | 0.3 | (0.0) |  | 702 | 1.4 | (0.1) |  | 434 | 0.8 | (0.1) |  | 202 | 0.4 | (0.0) |
| High income | 1550 | 1.6 | (0.1) |  | 646 | 0.7 | (0.0) |  | 267 | 0.3 | (0.0) |  | 2893 | 3.2 | (0.1) |  | 1388 | 1.6 | (0.1) |  | 565 | 0.6 | (0.0) |
| Total sample | 2096 | 1.4 | (0.0) |  | 935 | 0.6 | (0.0) |  | 424 | 0.3 | (0.0) |  | 3595 | 2.6 | (0.1) |  | 1822 | 1.3 | (0.0) |  | 767 | 0.5 | (0.0) |
|  |  |  |  |  |  |  |  |  |  |  |  |  |  |  |  |  |  |  |  |  |  |  |  |

**II. Prevalence of non-excessive GAD as a proportion of all broadly-defined GAD cases**

|  | **Non-excessive/**  **All GAD cases** | | |
| --- | --- | --- | --- |
|  | **Lifetime** | **12-month** | **30-day** |
| **Country** | **%** | **%** | **%** |
| Low/middle Income | 44.3 | 40.3 | 42.6 |
| High income | 33.6 | 29.5 | 29.8 |
| Total sample | 36.0 | 32.3 | 33.7 |
| Low/middle vs. high income χ^2^_1_ | 27.9 | 18.7 | 10.9 |
| (*p*) | (<.001) | (<.001) | (.001) |
|  |  |  |  |

**III. Persistence**

|  | **Non-excessive** | | | | |  | **Excessive** | | | | |
| --- | --- | --- | --- | --- | --- | --- | --- | --- | --- | --- | --- |
|  | **12-month/Lifetime** | |  | **30-day/12-month** | |  | **12-month/Lifetime** | |  | **30-day/12-month** | |
| **Country** | **%** | **(*SE*)** |  | **%** | **(*SE*)** |  | **%** | **(*SE*)** |  | **%** | **(*SE*)** |
| Low/middle Income | 52.0 | (2.0) |  | 52.1 | (2.9) |  | 61.4 | (1.7) |  | 47.3 | (2.1) |
| High income | 40.1 | (1.2) |  | 39.6 | (1.9) |  | 48.4 | (1.1) |  | 39.2 | (1.3) |
| Total sample | 43.4 | (1.0) |  | 43.7 | (1.6) |  | 50.9 | (0.9) |  | 41.1 | (1.1) |
| Low/middle vs. high income χ^2^_1_ (*p*) | 15.5 | (<.001) |  | 7.3 | (.007) |  | 23.8 | (<.001) |  | 7.1 | .008) |
| Non-excessive vs. Excessive χ^2^_1_ (*p*) | 26.9 | (<.001) |  | <0.1 | (.977) |  |  |  |  |  |  |
|  | | | | | | | | | | | |

^a^ Persistence was estimated indirectly by calculating the proportion of respondents with 12-month GAD among those with the lifetime disorder, and the proportion of respondents with 30-day GAD among those with the 12-month disorder. All chi-square tests controlled for age at interview and sex. The non-excessive vs. excessive chi-square test additionally controlled for country.

| **Table S3. Severity^a^ of non-excessive and excessive *DSM-5* GAD** | | | | | |
| --- | --- | --- | --- | --- | --- |
|  | | | | | |
|  | **Non-excessive** | |  | **Excessive** | |
| **GAD severity score** | **%** | **(*SE*)** |  | **%** | **(*SE*)** |
| 0 | 49.5 | (1.3) |  | 33.9 | (1.0) |
| 1 | 29.6 | (1.2) |  | 33.3 | (1.0) |
| 2 | 14.0 | (0.9) |  | 20.6 | (0.8) |
| 3 | 6.9 | (0.7) |  | 12.1 | (0.6) |
| χ^2^_3_ (*p*) | 28.9 | (<.001) |  |  |  |
|  | | | | | |

^a^ Values represent the proportion of respondents with non-excessive (*n* = 2,096) and excessive (*n* = 3,595) lifetime GAD, respectively, who met 0, 1, 2, or 3 of the three dichotomous indicators of GAD severity: frequent difficulty controlling worry, severe emotional distress due to worry, and marked functional impairment caused by worry. The chi-square test comparing the two groups’ severity distributions controlled for age at interview, sex, and country.

| **Table S4. Percentage^a^ of respondents with no GAD, non-excessive GAD, and excessive**  **GAD who subsequently developed each comorbid *DSM-IV* disorder** | | | | | | | | |
| --- | --- | --- | --- | --- | --- | --- | --- | --- |
|  | | | | | | | | |
|  | **No GAD** | |  | **Non-excessive** | |  | **Excessive** | |
| ***DSM-IV* disorder** | **%** | **(*SE*)** |  | **%** | **(*SE*)** |  | **%** | **(*SE*)** |
| Anxiety disorder |  |  |  |  |  |  |  |  |
| Panic disorder^b^ | 1.5 | (0.0) |  | 7.2 | (0.7) |  | 15.6 | (1.0) |
| Agoraphobia^b^ | 1.3 | (0.1) |  | 3.1 | (0.5) |  | 8.1 | (0.6) |
| Social phobia^b,c^ | 3.9 | (0.1) |  | 12.4 | (1.2) |  | 34.8 | (1.2) |
| Specific phobia^b,d^ | 7.6 | (0.1) |  | 18.3 | (1.2) |  | 31.9 | (1.1) |
| Posttraumatic stress disorder^e^ | 3.0 | (0.1) |  | 19.2 | (1.2) |  | 22.6 | (1.0) |
| Separation anxiety disorder^e,f^ | 2.8 | (0.1) |  | 10.9 | (1.1) |  | 17.5 | (1.0) |
| Any anxiety disorder^e^ | 12.9 | (0.2) |  | 41.7 | (1.5) |  | 62.3 | (1.1) |
| Mood disorder |  |  |  |  |  |  |  |  |
| Major depressive disorder^b^ | 10.0 | (0.1) |  | 48.0 | (1.6) |  | 54.5 | (1.2) |
| Bipolar spectrum disorder^b,g^ | 1.8 | (0.1) |  | 7.2 | (1.0) |  | 14.7 | (0.9) |
| Any mood disorder^e^ | 11.4 | (0.1) |  | 53.8 | (1.6) |  | 66.4 | (1.1) |
| Substance use disorder |  |  |  |  |  |  |  |  |
| Alcohol abuse or dependence^e^ | 9.3 | (0.1) |  | 16.7 | (1.3) |  | 23.3 | (1.0) |
| Drug abuse or dependence^e,h^ | 2.9 | (0.1) |  | 6.6 | (0.8) |  | 13.7 | (0.8) |
| Any substance use disorder^e^ | 10.3 | (0.2) |  | 18.9 | (1.3) |  | 26.7 | (1.0) |
| Disruptive behavior disorder | | |  |  |  |  |  |  |
| Attention-deficit/hyperactivity disorder^e,i,n^ | 2.0 | (0.1) |  | 7.2 | (1.5) |  | 11.1 | (1.3) |
| Oppositional defiant disorder^e,j,n^ | 2.9 | (0.2) |  | 10.2 | (1.6) |  | 13.4 | (1.4) |
| Conduct disorder^e,k,n^ | 2.2 | (0.1) |  | 7.1 | (1.3) |  | 10.7 | (1.3) |
| Intermittent explosive disorder^b,l^ | 3.0 | (0.1) |  | 8.5 | (1.7) |  | 18.1 | (1.4) |
| Bulimia nervosa^e,m^ | 0.7 | (0.0) |  | 1.9 | (0.5) |  | 4.9 | (0.6) |
| Binge eating disorder^e,m^ | 3.1 | (0.1) |  | 8.3 | (1.4) |  | 14.1 | (1.1) |
| Any disruptive behavior disorder^e^ | 4.3 | (0.1) |  | 9.8 | (1.1) |  | 16.8 | (0.8) |
| Any comorbid disorder^e^ | 28.2 | (0.2) |  | 73.7 | (1.5) |  | 87.1 | (0.7) |
|  | | | | | | | | |

^a^ Percentages reflect the proportion of respondents with no lifetime GAD, non-excessive GAD, and excessive GAD based on *DSM-5* criteria who also qualified for the lifetime *DSM-IV* disorder in each row.

^b^ Analyses were performed in the Part I sample. Mutually exclusive groups with non-excessive (*n* = 2,096) and excessive (*n* = 3,595) GAD were each compared with respondents having no lifetime GAD (*n* = 127,923), and then compared with each other.

^c^ Not assessed in Israel.

^d^ Not assessed in Australia, Israel, South Africa, or Ukraine.

^e^ Analyses were performed in the Part II sample, which included 1,983 respondents with non-excessive GAD, 3,418 respondents with excessive GAD, and 70,749 respondents with no lifetime GAD. The Part II sample for childhood disorders included 845 respondents with non-excessive GAD, 1,823 respondents with excessive GAD, and 38,776 respondents with no lifetime GAD.

^f^ Not assessed in Australia, Israel, Japan, New Zealand, Poland, South Africa, or Ukraine.

^g^ Includes bipolar I disorder, bipolar II disorder, or subthreshold bipolar disorder as defined by Merikangas et al. (2007). Not assessed in Belgium, France, Germany, Italy, Netherlands, South Africa, Spain, or Ukraine.

^h^ Not assessed in Portugal.

^i^ Not assessed in Australia, Bulgaria, Israel, Japan, New Zealand, South Africa, or Ukraine.

^j^ Not assessed in Australia, Bulgaria, Bulgaria 2, Israel, Japan, Lebanon, New Zealand, South Africa, or Ukraine.

^k^ Not assessed in Australia, Israel, Japan, Lebanon, New Zealand, South Africa, or Ukraine.

^l^ Not assessed in Australia, Belgium, Bulgaria 2, France, Germany, Israel, Italy, Medellin, Mexico, Murcia, Netherlands, Spain, or New Zealand.

^m^ Eating disorders were assessed in a random 50% of the Part II sample. Not assessed in Australia, Bulgaria, Bulgaria 2, Israel, Japan, Lebanon, Romania, South Africa, or Ukraine.

^n^ Recall of childhood disorders was restricted to respondents < 45 years of age at interview (with the exception of Ukraine, which was restricted to age < 40 at interview).

| **Table S5. Associations^a^ of non-excessive and excessive *DSM-5* GAD with the persistence of subsequent *DSM-IV* disorders** | | | | | | | | | | | |
| --- | --- | --- | --- | --- | --- | --- | --- | --- | --- | --- | --- |
|  | | | | | | | | | | | |
|  |  |  |  |  |  |  | **Excessive vs. Non-excessive** | | | | |
|  | **Non-excessive** | |  | **Excessive** | |  | **Unadjusted** | |  | **Adjusted** | |
| ***DSM-IV* disorder** | **RR** | **(95% CI)** |  | **RR** | **(95% CI)** |  | **RR** | **(95% CI)** |  | **RR** | **(95% CI)** |
| Anxiety disorder |  |  |  |  |  |  |  |  |  |  |  |
| Panic disorder^b^ | 1.1 | (0.9–1.3) |  | 1.0 | (0.8–1.2) |  | 0.9 | (0.7–1.1) |  | 0.9 | (0.7–1.1) |
| Agoraphobia^b^ | 1.2 | (0.8–1.9) |  | 1.4* | (1.2–1.7) |  | 1.2 | (0.7–1.9) |  | 1.2 | (0.7–1.8) |
| Social phobia^b,c^ | 1.4* | (1.2-1.8) |  | 1.2* | (1.1-1.3) |  | 0.8 | (0.7-1.0) |  | 0.8 | (0.7-1.0) |
| Specific phobia^b,d^ | 1.2 | (1.0-1.4) |  | 1.1 | (0.9-1.2) |  | 0.9 | (0.7-1.1) |  | 0.9 | (0.7-1.1) |
| Posttraumatic stress disorder^e^ | 1.2* | (1.1-1.4) |  | 1.3* | (1.1-1.4) |  | 1.0 | (0.9-1.2) |  | 1.0 | (0.9-1.2) |
| Separation anxiety disorder^e,f^ | 1.0 | (0.6-1.9) |  | 1.2 | (0.9-1.7) |  | 1.2 | (0.6-2.4) |  | 1.1 | (0.5-2.4) |
| Any anxiety disorder^e^ | 1.1* | (1.0-1.3) |  | 1.2* | (1.1-1.3) |  | 1.0 | (0.9-1.2) |  | 1.0 | (0.9-1.2) |
| Mood disorder |  |  |  |  |  |  |  |  |  |  |  |
| Major depressive disorder^b^ | 1.1 | (1.0-1.3) |  | 1.2* | (1.1-1.3) |  | 1.1 | (0.9-1.2) |  | 1.1 | (0.9-1.2) |
| Bipolar spectrum disorder^b,g^ | 1.0 | (0.8-1.3) |  | 1.1 | (1.0-1.3) |  | 1.2 | (0.9-1.6) |  | 1.1 | (0.8-1.5) |
| Any mood disorder^e^ | 1.1 | (1.0-1.2) |  | 1.2* | (1.1-1.3) |  | 1.1 | (1.0-1.2) |  | 1.1 | (1.0-1.2) |
| Substance use disorder |  |  |  |  |  |  |  |  |  |  |  |
| Alcohol abuse or dependence^e^ | 1.3 | (0.8-1.9) |  | 1.4* | (1.1-1.9) |  | 1.1 | (0.7-1.9) |  | 1.1 | (0.7-1.8) |
| Drug abuse or dependence^e,h^ | 1.3 | (0.8-2.2) |  | 1.5* | (1.1-2.1) |  | 1.2 | (0.6-2.2) |  | 1.1 | (0.6-2.1) |
| Any substance use disorder^e^ | 1.4 | (1.0-1.9) |  | 1.4* | (1.1-1.8) |  | 1.1 | (0.7-1.6) |  | 1.0 | (0.7-1.5) |
| Disruptive-behavior disorder |  |  |  |  |  |  |  |  |  |  |  |
| Attention-deficit/hyperactivity disorder^e,i,n^ | 1.5* | (1.0-2.3) |  | 1.6* | (1.1-2.4) |  | 1.1 | (0.6-1.9) |  | 1.1 | (0.6-1.9) |
| Oppositional defiant disorder^e,j,n^ | 0.8 | (0.1-8.9) |  | 1.8 | (0.8-4.0) |  | 2.2 | (0.2-27.0) |  | 0.3 | (0.0-4.2) |
| Conduct disorder^e,k,n^ | 1.2 | (0.1-14.5) |  | 1.1 | (0.3-4.0) |  | 1.0 | (0.1-16.8) |  | 1.4 | (0.1-38.0) |
| Intermittent explosive disorder^b,l^ | 1.0 | (0.8-1.4) |  | 1.0 | (0.8-1.3) |  | 1.0 | (0.7-1.5) |  | 1.0 | (0.7-1.5) |
| Bulimia nervosa^e,m^ | 0.6 | (0.1-2.3) |  | 1.0 | (0.6-1.7) |  | 1.7 | (0.4-7.8) |  | 1.9 | (0.4-9.2) |
| Binge eating disorder^e,m^ | 0.6 | (0.3-1.2) |  | 0.9 | (0.7-1.3) |  | 1.5 | (0.7-3.0) |  | 1.3 | (0.7-2.7) |
| Any disruptive behavior disorder^e^ | 1.0 | (0.7-1.3) |  | 1.1 | (0.9-1.3) |  | 1.1 | (0.8-1.7) |  | 1.1 | (0.8-1.6) |
| Any comorbid disorder^e^ | 1.3* | (1.2-1.5) |  | 1.3* | (1.2-1.4) |  | 1.0 | (0.9-1.1) |  | 1.0 | (0.9-1.1) |
|  | | | | | | | | | | | |

^a^ The risk ratios (RRs) come from person-level models estimating the association between variably-defined GAD and the persistence of a temporally secondary disorder, operationalized as the presence of a 12-month disorder among respondents with a lifetime history of that disorder. All models controlled for age of disorder onset, years since disorder onset, sex, and country. Adjusted models additionally controlled for severity, which was a 0–3 score summing across the three dichotomous indicators of uncontrollability, distress, and impairment.

^b^ Analyses were performed in the Part I sample. Mutually exclusive groups with non-excessive (*n* = 2,096) and excessive (*n* = 3,595) GAD were each compared with respondents having no lifetime GAD (*n* = 127,923), and then compared with each other.

^c^ Not assessed in Israel.

^d^ Not assessed in Australia, Israel, South Africa, or Ukraine.

^e^ Analyses were performed in the Part II sample, which included 1,983 respondents with non-excessive GAD, 3,418 respondents with excessive GAD, and 70,749 respondents with no lifetime GAD. The Part II sample for childhood disorders included 845 respondents with non-excessive GAD, 1,823 respondents with excessive GAD, and 38,776 respondents with no lifetime GAD.

^f^ Not assessed in Australia, Israel, Japan, New Zealand, Poland, South Africa, or Ukraine.

^g^ Includes bipolar I disorder, bipolar II disorder, or subthreshold bipolar disorder as defined by Merikangas et al. (2007). Not assessed in Belgium, France, Germany, Italy, Netherlands, South Africa, Spain, or Ukraine.

^h^ Not assessed in Portugal.

^i^ Not assessed in Australia, Bulgaria, Israel, Japan, New Zealand, South Africa, or Ukraine.

^j^ Not assessed in Australia, Bulgaria, Bulgaria 2, Israel, Japan, Lebanon, New Zealand, South Africa, or Ukraine.

^k^ Not assessed in Australia, Israel, Japan, Lebanon, New Zealand, South Africa, or Ukraine.

^l^ Not assessed in Australia, Belgium, Bulgaria 2, France, Germany, Israel, Italy, Medellin, Mexico, Murcia, Netherlands, New Zealand, or Spain.

^m^ Eating disorders were assessed in a random 50% of the Part II sample. Not assessed in Australia, Bulgaria, Bulgaria 2, Israel, Japan, Lebanon, Romania, South Africa, or Ukraine.

^n^ Recall of childhood disorders was restricted to respondents < 45 years of age at interview (with the exception of Ukraine, which was restricted to age < 40 at interview).

* Significant at the .05 level.

| **Table S6. Associations^a^ of non-excessive and excessive *DSM-5* GAD with the persistence of subsequent suicide-related outcomes** | | | | | | | | | | | |
| --- | --- | --- | --- | --- | --- | --- | --- | --- | --- | --- | --- |
|  | | | | | | | | | | | |
|  |  |  |  |  |  |  | **Excessive vs. Non-excessive** | | | | |
|  | **Non-excessive** | |  | **Excessive** | |  | **Unadjusted** | |  | **Adjusted** | |
| **Outcome** | **RR** | **(95% CI)** |  | **RR** | **(95% CI)** |  | **RR** | **(95% CI)** |  | **RR** | **(95% CI)** |
| Suicidal ideation | 1.4* | (1.1-1.8) |  | 1.6* | (1.3-1.8) |  | 1.1 | (0.9-1.4) |  | 1.0 | (0.8-1.4) |
| Suicide plan | 1.0 | (0.7-1.3) |  | 1.1 | (0.9-1.2) |  | 1.1 | (0.8-1.5) |  | 1.1 | (0.8-1.5) |
| Suicide attempt | 0.7 | (0.4-1.2) |  | 0.8 | (0.6-1.2) |  | 1.2 | (0.6-2.3) |  | 1.2 | (0.6-2.4) |
|  | | | | | | | | | | | |

^a^ The risk ratios (RRs) come from person-level analyses performed in the Part II sample,which included 1,983 respondents with non-excessive GAD, 3,418 respondents with excessive GAD, and 70,749 respondents with no lifetime GAD. Each model estimated the association between variably-defined GAD and the persistence of a temporally secondary suicide-related outcome, operationalized as the presence of 12-month suicidality among respondents with lifetime suicidality. The models for suicide plan were estimated among respondents with lifetime suicide plan and 12-month suicidal ideation. The models for suicide attempt were estimated among respondents with lifetime suicide attempt and 12-month suicidal ideation, and also controlled for lifetime suicide plan status. All models controlled for age of onset of the suicide outcome, years since onset of the suicide outcome, sex, and country. Adjusted models additionally controlled for severity, which was a 0–3 score summing across the three dichotomous indicators of uncontrollability, distress, and impairment.

* Significant at the .05 level.

| **Table S7. Treatment-seeking for GAD symptoms^a^ among respondents with non-excessive and excessive *DSM-5* GAD** | | | | | | | | | | | |
| --- | --- | --- | --- | --- | --- | --- | --- | --- | --- | --- | --- |
|  | | | | | | | | | | | |
|  |  |  |  |  |  |  | **Excessive vs. Non-excessive** | | | | |
|  | **Non-excessive** | |  | **Excessive** | |  | **Unadjusted** | |  | **Adjusted** | |
| **Outcome** | **%** | ***(SE)*** |  | **%** | ***(SE)*** |  | **χ^2^_1_** | ***p*** |  | ** χ^2^_1_** | ***p*** |
| Lifetime treatment^b^ | 43.4 | (1.7) |  | 51.6 | (1.3) |  | 6.8 | .009 |  | 2.7 | .101 |
| 12-month treatment^c^ | 18.9 | (1.2) |  | 26.9 | (1.2) |  | 19.6 | <.001 |  | 12.6 | <.001 |
|  | | | | | | | | | | | |

^a^ Values represent the proportion of respondents with broadly-defined GAD in the Part II sample who sought treatment specifically for GAD symptoms. All chi-square tests controlled for age at interview, sex, and country. Adjusted tests additionally controlled for severity, which was a 0–3 score summing across the three dichotomous indicators of uncontrollability, distress, and impairment.

^b^ Proportion of non-excessive (*n* = 1,542) and excessive (*n* = 2,657) lifetime cases reporting lifetime treatment for GAD.

^c^ Proportion of non-excessive (*n* = 689) and excessive (*n* = 1340) 12-month cases reporting 12-month treatment for GAD.

**Figure S1. Cumulative age-of-onset distributions and smoothed hazard rates for non-excessive and excessive *DSM-5* GAD**

Smoothed Hazard Rate, Non-excessive

Smoothed Hazard Rate, Excessive

Cumulative Incidence, Non-excessive

Cumulative Incidence, Excessive

**Supplement Reference**

Merikangas, K. R., Akiskal, H. S., Angst, J., Greenberg, P. E., Hirschfeld, R. M., Petukhova, M., & Kessler, R. C. (2007). Lifetime and 12-month prevalence of bipolar spectrum disorder in the National Comorbidity Survey replication. *Archives of General Psychiatry*, 64(5), 543–552. doi:10.1001/archpsyc.64.5.543
